# Detection of Senecavirus A in pigs from a historically negative national swine herd and associated with feed imports from endemically infected countries

**DOI:** 10.1111/tbed.14684

**Published:** 2022-08-28

**Authors:** Scott Dee, Karyn Havas, Gordon Spronk

**Affiliations:** ^1^ Pipestone Applied Research Pipestone Veterinary Services Pipestone Minnesota USA

**Keywords:** feed, imported, ingredients, naïve, Senecavirus A, swine

1

Since the initial report of the ability of contaminated feed to transmit porcine epidemic diarrhoea virus (Dee et al., 2014), further proof regarding the risk of feed and feed ingredients to the spread of viruses—including African swine fever virus, classical swine fever virus, foot and mouth disease virus, and pseudorabies virus—has been published (Niederwerder et al., 2019; Stenfeldt et al., 2022; Stoian et al., 2020). Another viral pathogen, Senecavirus A (SVA), also has been shown to survive well in feed, and can be transmitted to pigs following natural feeding behaviour (Caserta et al., 2022; Dee et al., 2018). As an example of its stability and long‐term infectivity in feed, SVA remained infectious to pigs following a 23‐day, 10,800‐km transport of virus‐positive feed across 29 states of the continental United States (Dee et al., 2022). SVA is a single‐stranded, non‐enveloped RNA virus within the same virus family as foot and mouth disease virus (FMDV) and can cause vesicular lesions in pigs that are visibly indistinguishable to those caused by FMDV (Joshi & Diel, 2015). Following observation of vesicles, once a differential diagnosis of SVA has been confirmed, the presence of SVA in pig populations does not prohibit the sale or export of pork meat and pork products. The risk of feed and feed ingredients for the spread of viruses of veterinary significance is a relatively new discovery; since it previously was thought not to occur, it has been ignored at the level of the classroom, the farm, government administration, global animal health organizations, and elected officials. Therefore, the purpose of this Research News is to describe the initial clinical diagnosis of SVA in a swine farm from a historically naïve national herd, and the results of a diagnostic investigation designed to identify potential routes of entry of SVA to the country and the farm. For confidentiality, names of all countries and companies will not be disclosed in this report.

In July 2022, vesicular lesions were observed on the snouts and feet of pigs in the case of a pork production system. Diagnostic testing indicated the presence of SVA in vesicular fluids and also ruled out the presence of FMDV. Prior to the onset of clinical signs, feed ingredients had been imported from other countries, several of which were known to be endemically infected with SVA, and formulated diets containing these imported ingredients were being fed to pigs prior to and during the onset of clinical signs. All feed ingredients had been stored in a warehouse at the feed mill, which was located at a separate site from the farm, and different personnel worked at the mill and at the farm. As this was the first case of SVA in the national swine herd, a diagnostic investigation was conducted to identify potential sources of viral entry. Published methods were used to detect viruses in dust samples collected from environmental surfaces at the affected farm, and dust samples and grain probe samples from feed ingredients and environmental surfaces at the feed mill (Dee et al., 2022; Gebhardt et al., 2022; Khanal et al., 2022). A total of 39 samples were collected, including eight dust samples from facilities and equipment, that is, floor surfaces from storage warehouses and associated driveways on affected farms and mills, along with feed mixers. In addition, 13 dust and probe samples were collected from bulk‐feed ingredients, including a mixture of soybean meal imported from one SVA‐positive and two SVA‐negative countries, along with raw soybeans and corn gluten meal from SVA‐negative countries. Regarding the soybean meal imports, the original shipment consisted of a mixture of 40,935 metric tons. At the time of the outbreak, 159 metric tons of the mixture remained in storage. Dust samples were collected from the surface of the meal and grain probe samples were collected from the interior of the meal. An additional 13 dust samples from micronutrients from SVA‐positive countries, including valine, lysine, methionine, vitamin C, threonine, and tryptophan, were sampled, along with a dust sample from the one tote bag containing lysine from an SVA‐positive country, due to the presence of visible debris, that is, feed dust and dirt on its external surface. Finally, four dust samples of poultry feed and dust from associated feed storage areas, together with samples of dust from plant food, were collected. Samples were tested for the presence of SVA RNA by PCR (Caserta et al., 2022) and results are summarized in Table 1.

**TABLE 1:** Summary of PCR testing of dust samples during SVA diagnostic investigation

Source of sample	Number of samples tested	Number of samples SVA (+)	Cycle threshold (Ct) value
Imported soybeans	5	0	38.0
Imported soybean meal: SVA positive country	5	2	31.5–35.0
Imported soybean meal: SVA negative countries	2	0	38.0
Imported corn gluten meal: SVA positive countries	1	0	38.0
Imported micronutrients: vitamins, amino acids, and minerals	13	0	38.0
Imported amino acid tote bag: surface dust	1	1	37.6
Surfaces: warehouse floor and driveway, feed mixer	8	0	38.0
Poultry: feed and storage facility	2	0	38.0
Powdered plant food	2	0	38.0
Positive controls: Soybean meal spiked with vesicular fluid from pigs	6	6	21.0–36.4

Across all samples tested, two of the five samples from soybean meal from a known SVA‐positive country, including a dust sample from the surface of the meal (Ct = 35.0) and a grain probe sample from the interior of the meal (Ct = 31.0), together with a dust sample from the external surface of the tote bag (Ct = 37.6), from a different SVA‐positive country, were found to be positive for SVA RNA. All remaining samples had a Ct value of 38 and were considered to be negative for SVA RNA. To validate the ability of the PCR assay to detect SVA RNA, six feed samples from an unaffected farm were spiked with vesicular fluid from affected pigs with clinical signs and previously confirmed to be SVA positive and FMDV negative. These samples were transported to a segregated area away from the affected farm using a designated vehicle for spiking and testing to avoid cross‐contamination. All six samples were PCR positive for SVA RNA.

This is the first report of the detection of SVA in feed ingredients imported from an endemically infected country that is potentially linked to cases of SVA in pigs from a historically negative national herd. Whilst sampling was limited, the diagnostic investigation evaluated many areas across the affected company's feed mill and farm facilities, and a wide variety of feed imports, including swine‐, poultry‐, and plant‐based ingredients used in the company. In addition, positive controls were used to validate the PCR assay's ability to detect SVA RNA in feed and feed ingredients, and to minimize the chances of false positive and false negative results. As this was the initial incursion of SVA in the country, the finding of SVA RNA only in specific types of samples, that is 40% of samples from imported soybean meal and one sample from surface debris obtained from an imported feed tote, is important. Also important is the grain probe sample collected from the interior of the residual 159 metric tons of soybean meal mixture which was a strong positive (Ct value of 31.0). Clinical signs of SVA were noted in pigs being fed diets formulated with the imported ingredients, suggesting that infectious SVA could have been present in the imported soybean meal. These results, combined with the many PCR‐negative samples from the affected production system, suggest that the specific ingredients that were SVA positive might have been the route of viral entry into the country, and cross‐contamination was well controlled, both at the sampling and laboratory levels. While it would have been helpful to collect more samples, this situation was considered an emergency, and the diagnostic investigation needed to be implemented immediately following observation of vesicular lesions.

This case describes the first potential link between the entry of a novel viral agent to a naïve national swine herd through the prior importation of feed ingredients from an endemically infected county. These results also support data from previous publications describing virus survival in feed and feed ingredients and the risk for domestic and transboundary disease spread conducted in experiments at the laboratory and demonstration project levels. These results should raise awareness throughout global agriculture that feed ingredients can serve as vehicles for the transboundary movement of viral pathogens and support the adoption of Responsible Imports practices to manage this risk (Patterson et al., 2019). Fortunately, this was SVA, and not FMDV; however, it might not be the case next time.

## CONFLICT OF INTEREST

The authors declare no conflict of interest.

2

## ETHICS STATEMENT

This report did not involve the use of experimental animals for research. Rather, it contains a summary of a diagnostic investigation conducted by employees from a specific production system who oversee the ethical raising of pigs in commercial facilities.

## Data Availability

The data are available on reasonable request from the corresponding author
